# Use of 360° virtual reality video in medical obstetrical education: a quasi-experimental design

**DOI:** 10.1186/s12909-021-02628-5

**Published:** 2021-04-10

**Authors:** Vera Arents, Pieter C. M. de Groot, Veerle M. D. Struben, Karlijn J. van Stralen

**Affiliations:** 1 Spaarne Gasthuis Academie, Spaarne Gasthuis Hospital, SpaarnePoort 1, 2134 TM Hoofddorp, the Netherlands; 2 Department of Gynaecology, Spaarne Gasthuis Hospital, Hoofddorp, the Netherlands

**Keywords:** Medical education, Virtual reality, Caesarean section

## Abstract

**Background:**

Video-based teaching has been part of medical education for some time but 360° videos using a virtual reality (VR) device are a new medium that offer extended possibilities. We investigated whether adding a 360° VR video to the internship curriculum leads to an improvement of long-term recall of specific knowledge on a gentle Caesarean Sections (gCS) and on general obstetric knowledge.

**Methods:**

Two weeks prior to their Obstetrics and Gynaecology (O&G) internship, medical students were divided in teaching groups, that did or did not have access to a VR-video of a gCS. Six weeks after their O&G internship, potentially having observed one or multiple real-life CSs, knowledge on the gCS was assessed with an open questionnaire, and knowledge on general obstetrics with a multiple-choice questionnaire. Furthermore we assessed experienced anxiety during in-person attendance of CSs, and we asked whether the interns would have wanted to attend more CSs in-person. The 360° VR video group was questioned about their experience directly after they watched the video. We used linear regression analyses to determine significant effects on outcomes.

**Results:**

A total of 89 medical students participated, 41 in the 360° VR video group and 48 in the conventional study group. Watching the 360° VR video did not result in a difference in either specific or general knowledge retention between the intervention group and the conventional study group. This was both true for the grade received for the internship, the open-ended questions as well as the multiple-choice questions and this did not change after adjustment for confounding factors. Still, 83.4% of the 360° VR video-group reported that more videos should be used in training to prepare for surgical procedures. In the 360° VR video-group 56.7% reported side effects like nausea or dizziness. After adjustment for the number of attended CSs during the practical internship, students in the 360° VR video-group stated less often (*p* = 0.04) that they would have liked to attend more CSs in-person as compared to the conventional study group.

**Conclusion:**

Even though the use of 360° VR video did not increase knowledge, it did offer a potential alternative for attending a CS in-person and a new way to prepare the students for their first operating room experiences.

**Supplementary Information:**

The online version contains supplementary material available at 10.1186/s12909-021-02628-5.

## Introduction

Video-based teaching has been part of medical education for a reasonable time [1–3]. An estimated 90% of the medical students have used videos (e.g. YouTube) to learn the different procedures and techniques before attending operations [4]. Watching educational videos has a positive effect on the learning process [5–8]; resulting in less study time required for study, better performance in comparison to students who only read text [9] and increased self-efficacy with regards to specific procedures [10]. Especially novices learn best by observation as compared to practicing, as all learning capacity is allocated to observing the correct procedure [11]. Watching videos has multiple advantages, including round-the-clock availability, access to more rare situations and information about complex interpersonal interactions, all without being present.

Commonly students watch operating videos on a computer, laptop, telephone or tablet, all on a 2D screen. However, today, more realistic options are available. With 360° Virtual Reality (VR) videos, the viewer has control over the viewing direction and has the possibility to stand in a 3D space. As this results in a more immersive experience than a traditional 2D video, this might lead to better learning outcomes [12].

Different studies have shown the benefits of 360° VR videos over traditional video in (medical) education [13–15]. It has been suggested that more immersive devices lead to better place illusions (feeling real) as compared to watching a video on a telephone [16]. One study suggested that students who had seen a 360° VR video performed better in performing a surgical knot, however a comparison group was lacking [17]. Research on whether it improves factual knowledge shows conflicting results. Some studies showed that there is much more involvement while watching the 360° VR video in comparison with 2D video, but that information retention did not differ [18–21].

In the medical curriculum, the use of 360° VR video could prepare students for attending new real-life situations. In a 6-week obstetrics and gynaecology (O&G) internship, students are able to attend one or more (gentle) caesarean sections (gCSs). However, this is not guaranteed since it depends on patient availability and patient preferences. Attending a CS can be impressive for a student and lead to anxiety and stress. Also as many caregivers are involved, and understanding everyone’s role and the specific organisation in the operation theatre can be perceived as complex.

We investigated if adding a 360° VR video prior to the internship curriculum leads to an improvement of long-term recall of specific knowledge about a gCS procedure and general knowledge about obstetrics. Furthermore we studied whether use of this video is appreciated by the students, whether it had an effect on anxiety during the first attendance of a CS in-person, and whether it could replace the attending of a CS in-person.

## Methods

### Procedure and participants

During the master phase of their medical education students of the Vrije University Amsterdam, the Netherlands, have to follow different practical internships for most of the medical specialisms. Prior their practical O&G internship of 6 weeks, they have a 2-week clinical training course (CTC) at one out of five different locations. After the 2-week CTC, the students have the practical part of their O&G internship in different hospitals over the region or abroad (so the CTC could be at location 1, and the practical part at location 2 etc). Apart from the internships abroad, assignments to the locations take place at random by the University. During their practical O&G internship they are supposed to attend one of more CSs. During a CSs they are present in the operating room as an observer and depending on the situation they can assist the surgeon.

We performed a prospective quasi-experimental study. To avoid a crossover effect, we used a temporal design by CTC location; first the complete CTC group did not see the video, and subsequently the complete CTC group did see the video. The moment the CTC groups did see the video differed between the 5 locations. In total, 18 CTC groups of on average eight O&G interns, were assigned to either; (1) the 360° VR video-group or (2) the conventional study group.

All students in the CTC groups were included in the study. A reminder was send after 1 and 3 weeks if the students did not complete the CS Knowledge Quiz. Students who took part in the study all agreed and signed informed consent. Participating students received a €7.50 gift voucher when they completed the CS Knowledge Quiz. According to Dutch law (Wet medisch-wetenschappelijk onderzoek met mensen) no formal ethical review was required. This study was performed according to the declaration of Helsinki and the study protocol and data analyses plan were approved by the Spaarne Gasthuis review board, approval October 15, 2018 (2018.0086).

### Conventional study group

Between September 2018 and November 2018, students from the CTC groups were enrolled for the conventional study group. They received the regular education program at their CTC location. They were approached after the O&G internship and were asked to complete a web-based electronic survey (CS Knowledge Quiz – Additional file 2 and below) about their knowledge of the CS procedure and their more general understanding of obstetrics.

### 360° VR video-group

From October 2018 to February 2019, the 360​​° VR video was part of the CTC for all students before the start of their O&G internship. During one of the educational sessions, the students could watch the 360° VR video. A researcher (VA) was present during the 360° VR video to allow for questions on the use of the device. Immediately after seeing the 360° VR video, the participants received a short questionnaire related to their satisfaction (VR 360° experience – Additional file 1 and below). After the O&G internship, they received the CS Knowledge Quiz.

### Video

**Fig. 1:**
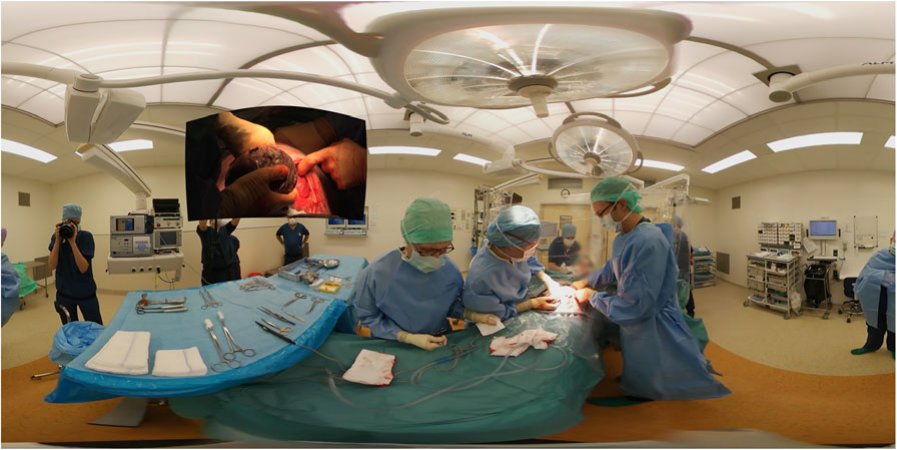
Snapshot from the 360 VR video

A 360​​° VR video of a gentle caesarean section [22] was made with permission of the patient. The 360​​° VR camera was positioned next to the main surgeon. The 360° VR video recorder gives a wide view of the whole theatre, so that the operation area was only a part of the total view, with a lack of details in the recordings, see Fig. 1. In the 360° video, it is not possible to zoom into the operation area. Therefore we installed a second (2D) camera at the foot of the bed, zoomed in at the operation area. It gave us a closer detailed vision of the operation. This 2D video view was simultaneously adapted into the 360° video view. A Dutch voice-over was added in which a gynaecologist (PdG) described the anatomical structures, the surgical technique, and gave information about the procedure, everyone’s function, organization, and situation. The duration of the 360​​° VR video was 27 min. The 360° VR video was shown on a VR headset (Oculus Go). Students could look in any direction of their own interest as well as pause and re-see the procedure when they wanted.

### Surveys – 360° VR video experience

Feedback related to the 360° VR video experiences, including perceived usefulness and side-effects, was obtained via a short survey (VR 360° experience, Additional file 1) consisting of 13 Likert-scale questions (from − 3 which means “strongly disagree” to + 3 which means “strongly agree”) and two open-end questions.

### Surveys – CS knowledge

After the 6 week O&G internship an online questionnaire was send to all students including eleven open ended questions and ten multiple choice questions (CS Knowledge, Additional file 2). The open ended questions aimed to get more insight into the specific knowledge about the procedure and organisation of a gentle CS. These open ended questions were developed and agreed on by two gynaecologists as being representative of the level of knowledge required for second-year master students, and tested by several volunteer medical students to evaluate the usefulness of the questions prior to the study. Manual coding of free text comments was performed for open ended survey responses; each item was awarded with maximal 10 points, this led to a sum score between 0 and 110 points, where higher points indicate better performance. There were three types of questions; “situation” relating to the situation in the operating theatre, “procedure” regarding tasks of various individuals in the room, relating to the aspects of the procedure itself and “organization”; which related to the organizational aspects of the gentle CS. Analyses were performed on both the total scores and the individual grouping scores.

To measure general knowledge about obstetrics ten multiple-choice questions, with four possible answers, were used. These questions were developed by the Progress Test Medicine, a national test organisation testing the progress of medical during their medical education (https://ivtg.nl/). Participants received 1 point for each correct answer and in total, a score between 0 and 10 could be achieved, where a higher score indicated better performance.

### Surveys – general information

General information was asked as part of the CS knowledge questionnaire. The grade of the internship before they participated in this study (psychiatric internship) was asked (grade 1–10), as a crude indication of the students general performance. Students were asked how their internship at the O&G department was graded (grade 1–10), as well as their anxiety level on a 7 point Likert scale (from − 3 which means “strongly disagree” to + 3 which means “strongly agree”) when they first were present at a CS procedure. It was also asked whether they had prior experience of VR, and whether they had watched other educational video’s. Finally, demographic information such as age and gender was collected.

### Statistical analysis

VA and KvS conducted a statistical analysis of the survey and internship grades using SPSS software. Continues variables are presented as medians and interquartile ranges (IQR), while nominal and ordinal variables are presented in percentages. Linear regression was performed while controlling for possible confounders (number of prior attended CS, number of CS attended during the internship, grade of previous internship, gender and the level of ambition to pursue a career in gynaecology, practiced progress test medicine questions before). *P*-values ​​smaller than .05 were considered statistically significant. To analyse the data, SPSS version 24 was used.

## Results

In total, 109 students attended the CTCs. Out of 56 CTC students who participated in the conventional education group, 48 (85.7%) completed the study. In the CTC groups who watched the 360° VR video-group, 53 students were present. From them, 41 (77%) completed the CS knowledge questionnaire and were included in the analysis.

Participants ages (mean 23 years), starting year of the bachelor (median 2014) and master (median 2017) were similar between the conventional education and 360° VR video groups, see Table 1. Most participants were female (62.9%) and 58.4% of the students had no experience with 360​​° VR videos before they participated in this study. Groups differed with respect to the previous VR experience (53.7% in the 360° VR video-group vs 31.3% in the conventional group), as well as the number of participants who had practiced gynaecology questions (22.0% in the 360° VR video-group vs 50% in the conventional group). Most participants (79.8%) had never been present, performed or assisted in a CS procedure before. A small portion (17%) of the participants did see a 2D video of a CS in the preceding period during and before their internship. In addition, participants who wanted to pursue a career as a gynaecologist used the VR glasses more often as compared to those who had not seen the video (Table 2).

**Table 1:** Demographic information on the students participating in the CS knowledge questionnaire

	Total *N* = 89	360° VR video-group *N* = 41	Conventional study group *N* = 48	*P* value
Age (mean, range)	23.5 (21–28)	23.6 (22–28)	23.3 (21–26)	0.24
Gender (% males)	33 (37.1%)	17 (41.5%)	16 (33.3%)	0.43
Year start Bachelor (median, range)	2014 (2012–2015)	2014 (2012–2015)	2014 (2012–2015)	0.56
Year start Master (median, range)	2017 (2015–2018)	2017 (2017–2018)	2017 (2015–2018)	0.82
Grade Psychiatry (mean, range)	7.85 (6–9)	7.76 (6–9)	7.94 (6–9)	0.19
Previous VR experience (yes,%)	37 (41.6%)	22 (53.7%)	15 (31.3%)	0.03
Number of CS attended before residency (mean, min - max)	0.26 (0–4)	0.27 (0–3)	0.26 (0–4)	0.96
Number of CS attended during residency (mean, min - max)	2.99 (0–14)	3.5 (1–11)	2.52 (0–14)	0.06
Viewed CS videos in the last 9 weeks (missing *n* = 1)	15 (16.9%)	4 (10.1%)	11 (22.69%)	0.11
Practice gynecology questions	33 (37.1%)	9 (22.0%)	24 (50.0%)	0.006

**Table 2:** 360° VR video experience among students in the 360° VR video group

	Strongly disagree *N (%)* *−3*	Disagree *N (%)* *−2*	Slightly disagree *N (%)* *− 1*	Neutral *N (%)* *0*	Slightly agree *N (%)* *1*	Agree *N (%)* *2*	Strongly agree *N (%)* *3*	*M (SD)*
*1. I found watching the VR video an inspiring experience.*	1 (1.9)	0 (0.0)	1 (1.9)	4 (7.5)	10 (18.9)	25 (47.2)	12 (22.6)	1.74 (1.15)
*2. Watching the VR video contributed to my learning experience/increased my knowledge, about the gentle caesarean section.*	1 (1.9)	0 (0.0)	0 (0.0)	3 (5.7)	12 (22.6)	26 (49.1)	11 (20.8)	1.77 (1.05)
*3. In comparison with normal/2D videos I was more actively involved while watching the VR video.*	1 (1.9)	2 (3.8)	2 (3.8)	8 (15.1)	9 (17.0)	19 (35.8)	12 (22.6)	1.40 (1.45)
*4. Watching the VR video was useful.*	0 (0.0)	0 (0.0)	0 (0.0)	0 (0.0)	13 (24.5)	30 (56.6)	10 (18.9)	1.94 (0.66)
*5. This method of teaching, the VR video, should be used in future CTCs or residencies.*	1 (1.9)	0 (0.0)	1 (1.9)	6 (11.3)	10 (18.9)	22 (41.5)	13 (24.5)	1.68 (1.21)
*6. This method of teaching, watching the VR video, was enjoyable.*	0 (0.0)	1 (1.9)	1 (1.9)	4 (7.5)	8 (15.1)	25 (47.2)	14 (26.4)	1.83 (1.09)
*7. I would like to use this teaching method, the VR video, to prepare for future surgical procedures.*	0 (0.0)	1 (1.9)	2 (3.8)	3 (5.7)	6 (11.3)	24 (45.3)	17 (32.1)	1.91 (1.15)
*8. The HMD was easy to use.*	1 (1.9)	0 (0.0)	1 (1.9)	2 (3.8)	6 (11.3)	27 (50.9)	16 (30.2)	1.96 (1.11)
*9. I did experience nausea, dizziness, or headache while viewing the VR video.*	10 (18.9)	12 (22.6)	1 (1.9)	5 (9.4)	13 (24.5)	8 (15.1)	4 (7.5)	−0.26 (2.04)
*10. I found the HMD comfortable to wear.*	0 (0.0)	9 (17.0)	6 (11.3)	8 (15.1)	11 (20.8)	14 (26.4)	5 (9.4)	0.57 (1.62)
*11. I found the quality of the videos and the audio to be excellent.*	0 (0.0)	6 (11.3)	3 (5.7)	5 (9.4)	16 (30.2)	17 (32.1)	6 (11.3)	1.00 (1.47)
*12. I could look around the operating room comfortably, without any discomfort.*	0 (0.0)	2 (3.8)	4 (7.5)	4 (7.5)	8 (15.1)	28 (52.8)	7 (13.2)	1.45 (1.26)
*13. I want to see more medical education VR videos.*	0 (0.0)	1 (1.9)	2 (3.8)	3 (5.7)	10 (18.9)	21 (39.6)	16 (30.2)	1.81 (1.16)

Note: Depending on the level of agreement scores were assigned ranging from − 3 for strongly disagree, via 0 for neutral, to + 3 for strongly agree. Mean scores were calculated from this number, suggesting positive numbers for a positive association, and negative numbers for a negative association. *N(%)* number of participants and percentage, *M* computed mean score, *SD* Standard deviation

### VR 360° experience

Tables 2 and 3 show the student’s feedback on the 360° VR video experiences. Nearly all students described the 360° VR video as useful (100%), fun (86.7%) and inspiring (90.0%). Most (83.4%) students reported that more 360° VR videos should be used in future courses and that it would be helpful to prepare future surgical procedures (93.3%). Twenty seven students (90%) reported that they would like to see more medical 360° VR videos, 76.7% felt more “actively involved” during the CS procedure in comparison to traditional videos. Most of the students (90%) reported that watching the 360° VR video improved their knowledge of a gentle CS. In terms of technical issues, 16 students (56.7%) experienced at least a little bit of dizziness, a headache or nausea while viewing the 360​​° VR video. Eight (26.7%) students reported that the VR device was not comfortable to wear. Almost all students (93.3%) reported that the VR device was easy to use, but 10% of the students reported that the quality (image and sound) of the 360° VR video was not excellent.

**Table 3:** Agreement of students in the 360° VR video-group and conventional group regarding various outcomes from the general information questionnaire

Item	Strongly disagree *N (%)*	Disagree *N (%)*	Slightly disagree *N (%)*	Neutral N (%)	Slightly agree N (%)	Agree N (%)	Strongly agree N (%)	
*I want to pursue a career as a gynecologist.*								
Total	15 (16.9)	23 (25.8)	4 (4.5)	16 (18.0)	23 (25.8)	6 (6.7)	2 (2.2)	*P* = 0.027
Control group	6 (14.6)	18 (37.5)	0 (0)	8 (16.7)	11 (22.9)	1 (2.1)	1 (2.1)	
Experimental group	9 (18.8)	5 (12.2)	4 (9.8)	8 (19.5)	12 (29.3)	5 (12.2)	1 (2.4)	
*During the gynecology internship I would have liked to attend more CSs.*								
Total	5 (5.6)	11 (12.4)	8 (9.0)	13 (14.6)	15 (16.9)	26 (29.2)	11 (12.4)	*P* = 0.088
Control group	0 (0.0)	5 (10.4)	5 (10.4)	5 (10.4)	13 (27.1)	14 (29.2)	6 (12.5)	
Experimental group	5 (12.2)	6 (14.6)	3 (7.3)	8 (19.5)	2 (4.9)	12 (29.3)	5 (12.2)	
*The first time I attended a CS during my gynecology residency I was (a bit) anxious.*								
Total	4 (4.5)	8 (9.0)	5 (5.6)	18 (20.2)	26 (29.2)	19 (21.3)	9 (10.1)	*P* = 0.62
Control group	1 (2.1)	4 (8.3)	3 (6.3)	11 (22.9)	15 (31.3)	8 (16.7)	6 (12.5)	
Experimental group	3 (7.3)	4 (9.8)	2 (4.9)	7 (17.1)	11 (26.8)	11 (26.8)	3 (7.3)	
*If a HMD had been present at the department during my residency, I would have watched (parts of) the VR video of the GCS again..*								
Experimental group	3 (7.3)	7 (17.1)	4 (9.8)	5 (12.2)	10 (24.4)	10 (24.4)	2 (4.9)	

Note: *N(%)* number of participants and percentage

### gCS knowledge

There was no significant effect of the 360​​° VR video on all different measurements of knowledge (Table 4). This was true for the grade received for the O&G internship (mean 7.75 in 360° VR group vs 7.83 among the conventional group). The results for the open-ended questions on the gCS were also similar, both overall (74.6 experimental, 73.6 conventional), as well as specific for the procedure, organisation and the situation (see Table 4). Knowledge on general obstetrics, as assessed via the multiple-choice questions was again similar between the groups. Adjustment for possible confounding factors did not change these results.

**Table 4:** Effect of the 360° VR video on obtained CS knowledge for the 360° VR video-group as compared to conventional education group

	360° VR video-group	*Conventional education*	*Difference*	*Difference, adjusted*	*p*
gCS specific questions (min and max scores)					
Total (0–110)	*74.6*	*73.6*	*0.91 (−3.28 to 5.11)*	*0.92 (−3.54 to 5.39)*	*0.66/0.68*
Procedure (0–50)	*35.0*	*34.9*	*−0.15 (−2.40 to 2.10)*	*−0.42 (−2.80 to 1.96)*	*0.89/0.73*
Organization (0–20)	*11.6*	*11.4*	*− 0.25 (− 2.2 to 1.68)*	*0.07 (− 2.02 to 2.16)*	*0.80/0.95*
Situation (0–40)	*28.4*	*27.0*	*1.32 (−1.12 to 3.75)*	*1.27(−1.40 to 3.95)*	*0.28/0.35*
General obstetric knowledge (Multiple choice questions (0–10)	*6.63*	*6.67*	*−0.033 (−.613 to 0.55)*	*− 0.081 (− 0.73 to 0.57)*	*0.91/0.80*
Grade internship Obstetics & gynecology (1–10)	*7.75*	*7.83*	*−0.08 (−.33 to .16)*	*−.142 (− 0.41 to 0.12)*	*0.49/0.28*

*Note:* M=mean, Mean difference = (experimental – control), adj. M = mean adjusted for possible confounding factors (e.g. level of ambition to pursue a career in gynaecology, number of CS attended before and during the residency, grade of previous residency and if they practiced VGT MC questions before), b = regression coefficient, 95%CI = 95% confidence interval, p = *p* value of the b, ^a^ = adjusted for possible confounding factors

**Fig. 2:**
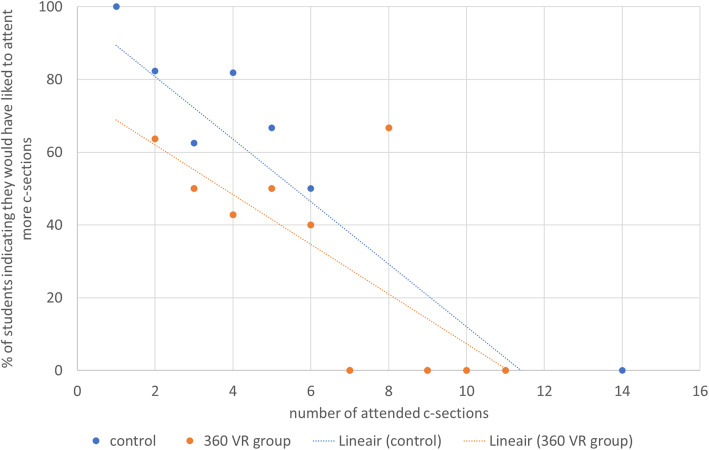
Association between the number of attended caesarean sections and the % of participants stating that they would like to attend more caesarean section

The median number of attended CS’s was 2 (IQR 1–4). Three participants (2 from the 360° VR video-group) had their internship in a hospital in Aruba, and attended many more CSs (8, 10, 14, and). The ten participants who attended six CS or more did not indicate that they would like to attend more CSs during their internship. From the 76 participants who attended 5 or less CS’s, 65.8% indicated that they would have liked to attend more CSs, which was inversely correlated with the number of attended CSs. Overall, more participants in the conventional study group (68.8%) indicated that they would have liked to attend more CS, as compared to the 360° VR video-group (46.4%, *p* = 0.09, Fig. 2) When we adjusted this comparison for the number of CSs they had attended, as well as whether they wanted to pursue a career in gynaecology, this difference was significant (*p* = 0.04). There was no significant difference between the two groups in anxiety the first time they visited a CS in-person (Table 3).

## Discussion

### Main findings & interpretation

Our first aim was to study whether the addition of an 360​​° VR video to the O&G internship curriculum leads to an improvement of long-term recall of specific knowledge on CS as well as general knowledge retention. We did not find a significant difference in the long-term recall of knowledge between the 360° VR video-group and those who only received conventional education.

Our second aim was to determine the appreciation by the students, whether it had an effect on anxiety during the first attendance of a CS in-person, and whether it could be an alternative for attending CSs in-person. We found no difference in the experienced stress/anxiety when they were present in the operating room during the first CS of their internship. However, feedback from participants was positive; the students reported that the 360​​° VR video was both entertaining and was being perceived as beneficial to learning. In addition, participants who had watched the 360​​° VR video indicated less often that they would have wanted to attend more CSs during their internship than participants who had not watched the video.

The COVID-19 outbreak and shortage of protection material has led in some places to allowing less students into the operating room, or only to watch from a distance. Furthermore, in the Netherlands, only 15% of the women deliver via a CS, and only half of them is planned [23]. For this reason it is not possible to guarantee a minimum number of CSs to be attended for by the students. Indeed two participants (2.2%) did not attend any CS and 31% of the participants attended only one. Therefore, the addition of a 360° VR video to the curriculum might provide an alternative educational option for those having limited opportunities to attend a sufficient number of CSs in-person.

It was highly ambitious to expect that adding one video of a CS would lead to better learning outcomes. However, previous research has shown that VR technology could positively influence students’ motivation and intention to engage in learning activities [24, 25]. Indeed the majority of the students who received VR teaching enjoyed it and recommended it for future courses as was also shown by others.^33^ We had hoped that when someone is motivated and engaged, he or she has a greater chance to learn effectively and retain knowledge [26–28].

### Strengths and limitations

There are several possible explanations for the lack of effect on learning; the students often indicated that they preferred the 2D detailed view, instead of the operation room and the general procedures. This may explain the lack of gained knowledge on the open questions regarding surgery. Furthermore, switching between two sources (general operation room, the surgical team and it’s procedures versus the surgical procedure itself) of information (known as a split-attention effect) may have reduced the learning capacity due to increased cognitive workload [11, 29].Therefore, it might have been better if we would have switched between scenes instead of providing all the information simultaneously. In addition, some studies [30, 31] have indicated that immersive technologies, such as 360​° VR videos, may distract users from the actual video content and thus further increasing the cognitive workload with less relevant use (Cognitive load theory). This could have been the case in the current research as most students (58%) reported that they did not have experience with VR before.

Sometimes learners get lost or are unable to navigate through the VR interface [32]. Therefore, there was a researcher present during the 360° VR video to allow for questions. However, we cannot rule out that students were lost in the VR interface. Finally, the fact that part of the students reported dizziness and/or mild nausea may have reduced the concentration of the students and therefore their learning capacity.

It is important to note that technology itself does not improve learning. The content is far more important. The students indicated that they would have liked to receive more theoretical information during the 360° VR video even though a voice-over was included in the movie (providing answers to nearly all open-ended questions that were in the questionnaire). However, it remains that the strength of the use of video over real-life provides the opportunity to emphasize or point out important information, which we might not have fully utilized.

There were several limitations to this study; first of all the CTC groups and thus the participants are not randomly assigned to either the conventional study or the experimental group. We did not want to have any cross-over effects, and therefore decided to have a temporal design. The CTC groups receive teaching according to the same protocol, but the practical part depends on the circumstances. Given that all students have equal chances to go to different locations, and this was independent of the VR video, we assume that the effect on our results will have been small. However, groups were not identical with respect to practicing gynaecology questions as well as the number of attended CSs. Even though we adjusted for these factors, we cannot rule out any residual confounding.

Secondly, students were not rewarded for good performance on the knowledge questions, nor did it count towards their grade. Therefore students may not have been fully motivated to answer the questions, however we do not believe that this will have differed between the groups. Finally, although 360° VR videos are widely regarded as a virtual reality, it should be noted that the location of the viewer is fixed and it is not possible to walk through, or interact with the environment.

## Conclusion

Although addition of the 360° VR video to the medical curriculum did not lead to better information retention it was highly appreciated by the medical students. It might provide an alternative for those who were not able to attend a CS in-person. Therefore, especially among situations where the number of procedures are unpredictable, such as a CS, addition of a 360​​° VR video to the curriculum can be a useful, low-cost tool to fulfil the learning needs of students.

## Supplementary Information

**Additional file 1.** Questions regarding the VR experience.

**Additional file 2.** Questions to test the knowledge of the medical students.

## Data Availability

The datasets used and analysed during the current study are available from the corresponding author on reasonable request. The video of the CS is not available for open use, as the women did not give permission for use outside education for students in the region.
