# Supplementary material for: Use of 360° virtual reality video in medical obstetrical education: a quasi-experimental design
Source: BMC Med Educ. 2021 Apr 10;21:202. doi: 10.1186/s12909-021-02628-5 (PMC8035054; doi:10.1186/s12909-021-02628-5)
Supplement: Supplementary file 1 — Additional file 1. Questions regarding the VR experience. [file 12909_2021_2628_MOESM1_ESM.docx]

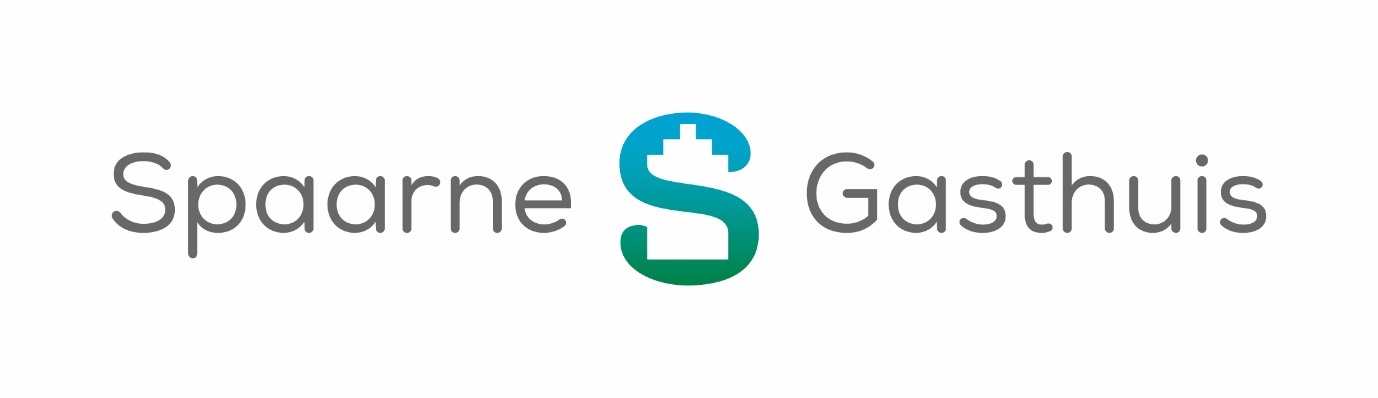
Appendix I Questions regarding the VR experience.

You just watched the VR-video of a gentle cesarean section. Here are 13 statements about the VR video. Could you please indicate how much you agree with each statement?

|  | Completely disagree | disagree | Partly disagree | Neutral | Partly agree | agree | Completely agree |
| --- | --- | --- | --- | --- | --- | --- | --- |
| I found watching the VR video an inspiring experience | 1 | 2 | 3 | 4 | 5 | 6 | 7 |
| Watching the VR video increased my learning experience or my knowledge on the gentle cesarean section | 1 | 2 | 3 | 4 | 5 | 6 | 7 |
| In comparison with normal/2D videos I was more actively involved in the VR video | 1 | 2 | 3 | 4 | 5 | 6 | 7 |
| Watching the VR video was useful | 1 | 2 | 3 | 4 | 5 | 6 | 7 |
| The VR video should be added to future KTO | 1 | 2 | 3 | 4 | 5 | 6 | 7 |
| This way of teaching – watching a VR video – was pleasant | 1 | 2 | 3 | 4 | 5 | 6 | 7 |
| I would like to use this way of teaching to prepare for future surgical procedures | 1 | 2 | 3 | 4 | 5 | 6 | 7 |
| The VR glasses were easy to use | 1 | 2 | 3 | 4 | 5 | 6 | 7 |
| I became nauseous, dizzy or got a headache from watching the VR video | 1 | 2 | 3 | 4 | 5 | 6 | 7 |
| I found the VR glasses/headset comfortable to wear | 1 | 2 | 3 | 4 | 5 | 6 | 7 |
| I found the quality of the image and sound excellent | 1 | 2 | 3 | 4 | 5 | 6 | 7 |
| I would look around in the operation theater without any inconvenience | 1 | 2 | 3 | 4 | 5 | 6 | 7 |
| I would like to watch more medical education VR videos | 1 | 2 | 3 | 4 | 5 | 6 | 7 |
