# Supplementary material for: Use of 360° virtual reality video in medical obstetrical education: a quasi-experimental design
Source: BMC Med Educ. 2021 Apr 10;21:202. doi: 10.1186/s12909-021-02628-5 (PMC8035054; doi:10.1186/s12909-021-02628-5)
Supplement: Supplementary file 2 — Additional file 2. Questions to test the knowledge of the medical students. [file 12909_2021_2628_MOESM2_ESM.docx]

Appendix II

Questions to test the knowledge of the medical students.

PART A. Multiple choice questions:

1. A 17- year-old primigravida gives birth á term to a son of 3600 grams via caesarean section. She has polyhydramnios caused by an oesophageal atresia. Directly after the section a severe haemorrhage post-partum (HPP) develops. In this case the main risk factor for HPP is
   1. Delivery per caesarean section
   2. Young maternal age
   3. Nulli-partiy
   4. Polyhydramnion
   5. I do not know
2. After birth, the gynaecologist inspects the cut umbilical cord for the number of blood vessels. Normally the umbilical cord has
   1. 3 vessels
   2. 2 vessels
   3. 4 vessels
   4. I do not know

- Onderkant formulier

Onderkant formulier

Onderkant formulier

Onderkant formulier

Onderkant formulier

Onderkant formulier

Onderkant formulier

1. In the case of a delivery, in which the child is in the normal position (back of the head), there is a non-progressive expulsion. The decision whether the child can be born with a vaginal delivery depends on the descent of the head. A vaginal birth is possible when the deepest part of the caput has descended to just past:
   1. Hodge 1
   2. Hodge 2Bovenkant formulier
   3. Hodge 3
   4. Hodge 4
   5. I do not know Onderkant formulier
2. Leopolds’ second handle is intended to:
   1. Determine the ballot of the front part
   2. Determine the height of the fundus
   3. Determine the position of the child’s back
   4. Determine the degree of descent of the present sectionBovenkant formulier

1. Stress and anxiety inhibit contractions during labor. Which mechanism in the muscles of the uterus plays the most important role in this?
   1. Closing the gap junctions between the muscle cells.
   2. Reduced contraction due to an increase in catechol amines.
   3. Reduced maturation of the muscle tissue.
   4. Decreased expression of oxytocin receptors.
   5. I do not know
2. Compared to a normal delivery, the chance of a fetal-maternal transfusion is NOT increased with
   1. Manual placenta removal;
   2. Caesarean section;
   3. Breech delivery;
   4. External version (attempt);
   5. I do not know.
3. During parturition of 25-year-old woman there appears to be a shoulder dystocia. Which of the following actions is then indicated first?
   1. A Caesarean section.
   2. A version and extraction.
   3. A vacuum extraction.
   4. Suprapubic impression.
   5. I do not know.
4. The average heart rate of a 32 week fetus is compared to that of 10 weeks. Relative to 32 weeks, the mean fetal heart rate at 10 weeks is, in the majority of cases:
   1. Equal;
   2. Higher;
   3. Lower;
   4. I do not know.
5. In a pregnant woman, the fundus uteri is palpated at the top of the navel. In case of a normal pregnancy, this fits with a term of:
   1. 24 weeks
   2. 28 weeks
   3. 32 weeks
   4. I do not know.

- Onderkant formulier

10. What is the lower and upper limit (expressed in amenorrhea) of the at term period of a pregnancy?

- 1. 35 and 40 weeks;
  2. 37 and 40 weeks;
  3. 35 and 42 weeks;
  4. 37 and 42 weeks;
  5. I do not know.

Correct answers 1d, 2a, 3c, 4c, 5b, 6c, 7d, 8b, 9a, 10d

PART B. open ended questions

4 questions are about the situation

Question 1. Which people are present at an OR during the gentle caesarean section?

Question 2. Name two tasks of the circulation operation assistant during a gentle caesarean section.

Question 3: Name two tasks of the nurse who is present during the OR during a gentle caesarean section

Question 4 Name three tasks of the anesthetist / anesthetist during a gentle caesarean section.

2 questions are about the organization

Question 5: Name three aspects in which a so-called gentle caesarean section differs from a normal caesarean section

Question 11: Name four aspects that are discussed during debriefing

5 questions are about the procedure

Question 6: which layers do you pass when performing a caesarean section?

Question 7: Which layers are opened with the scalpel?

Question 8: Which layers are closed with suture in a caesarean section?

Question 9: Name 4 indications for a caesarean section?

Question 10: Name 4 potential complications for the mother of a caesarean section
